# Changes in the Constitution of the Southern Dental Association, Making It a Branch of the National Dental Association

**Published:** 1898-06

**Authors:** 


### PROCEEDINGS.

### Changes in the Constitution of the Southern Dental Association, Making it a Branch of the National Dental Association.

REVISION OF THE CONSTITUTION.

The Committee on Revision of the Constitution of "The Southern Dental Association" which, as last revised, was adopted in 1895, reported a very thorough revision, adapting it to the government of "The Southern Branch of the National Dental Association," the name adopted by the new organization. The most radical changes are embodied in the adoption of the delegate system of membership, as set forth in Articles III. and IV. of the Constitution of the National Association.

The list of Committees was considerably extended, Materia Medica and Cataphoresis being added to "Sec. 6, on Pathology and Therapeutics;" Bacteriology was added to "Sec. 7, on Histology and Microscopy;" Metallurgy added to "Sec. 8, on Chemistry;" Nomenclature added to "Sec. 9, on Literature and Voluntary Essays." The following new sections were also added: "14, Anatomy;" "15, Orthodontia;" "16, Oral Surgery;" Article XIV. of the Constitution of the National, and also the "Standing Resolutions" in force in the latter, were appended to the Constitution of the Southern Branch. This includes all the more important features embodied in the revision of the Constitution of the old Southern Association, in making it conformable to the Constitution of the National Association. The revision received very full discussion and was adopted without opposition.

AMENDMENT TO THE PATENT LAWS.

A communication was read from Dr. R. Ottolengui, chairman of a committee of the Dental Society of the State of New York, relative to a proposed amendment to the patent laws, relative to processes or methods of treating human disease, or methods of restoring or replacing lost parts of the human body. A list of seven patients was given covering methods of restoring lost portions of the teeth; the list includes laboratory-made fillings to be cemented into cavities; inserting screws to secure fillings; swaging a partial cap with pins on the inner side, and other methods in daily use. The matter was referred to a committee, whose report at a later session was discussed at some length and a resolution adopted endorsing the proposed amendment.

